# Sex differences in children's health status as measured by the Pediatric Quality of Life Inventory (PedsQL)™: cross-sectional findings from a large school-based sample in the Netherlands

**DOI:** 10.1186/s12887-021-03059-3

**Published:** 2021-12-18

**Authors:** Annelieke Hijkoop, Chantal A. ten Kate, Marlous J. Madderom, Hanneke IJsselstijn, Julie A. Reuser, Hendrik Koopman, Joost van Rosmalen, André B. Rietman

**Affiliations:** 1 Department of Paediatric Surgery and Intensive Care, Erasmus MC Sophia Children’s Hospital, Rotterdam, the Netherlands; 2 Department of Developmental and Educational Psychology, Faculty of Social Sciences, Leiden University, Leiden, the Netherlands; 3 Department of Clinical Psychology, Faculty of Social Sciences, Leiden University, Leiden, The Netherlands; 4 Department of Biostatistics, Erasmus MC, Rotterdam, the Netherlands; 5 Department of Epidemiology, Erasmus MC, Rotterdam, the Netherlands; 6 Department of Child and Adolescent Psychiatry/Psychology, Erasmus MC Sophia Children’s Hospital, Rotterdam, The Netherlands

**Keywords:** Health status, Health-related quality of life, PedsQL, Sex differences, Children

## Abstract

**Background:**

Previous research has shown that female adolescents and adults report lower health status than their male peers. Possibly, this discrepancy already develops during childhood. We collected sex-specific data with the Pediatric Quality of Life Inventory (PedsQL) in a large school-based sample.

**Methods:**

The online version of the PedsQL was administered to healthy Dutch children aged 5–7 years (parent proxy-report), 8–12 years (parent proxy-report and child self-report), and 13–17 years (parent proxy-report and child self-report), recruited through regular primary and secondary schools. Sex differences were assessed using t-tests or Mann–Whitney U-tests. Wilcoxon signed-rank tests and intraclass correlation coefficients served to compare parent proxy-reports with child self-reports. Multivariable linear regression analyses were used to assess the associations of sex of the child, age, and parental educational level with PedsQL scores.

**Results:**

Eight hundred eighty-two parents and five hundred eighty one children were recruited from 15 different schools in the Netherlands. Parents of 8-to-12-year-olds reported higher scores on School Functioning for girls than for boys (mean difference [MD]: 6.56, p < 0.001). Parents of 13-to-17-year-olds reported lower scores on Physical and Emotional Functioning for girls than for boys (MDs: 2.14 and 5.79, p = 0.014 and p < 0.001, respectively). Girls aged 8–12 years reported lower scores than boys in this age group on Physical Functioning (MD: 3.09, p = 0.005). Girls aged 13–17 years reported lower scores than boys in this age group on Physical Functioning (MD: 3.67, p < 0.001), Emotional Functioning (MD: 8.11, p < 0.001), and the Total Score (MD 3.26, p = 0.004). No sex differences were found in children aged 5–7 years. Agreement between child self-reports and parent proxy-reports was poor to moderate**.**

**Conclusions:**

Girls generally had lower PedsQL scores than boys, both in parent proxy-reports and in child self-reports. We recommend to apply sex-specific data when assessing health status using the PedsQL.

**Supplementary Information:**

The online version contains supplementary material available at 10.1186/s12887-021-03059-3.

## Background

The quality of pediatric healthcare has improved and mortality rates have declined over the last decades [1, 2]. An increasing number of children are living with a chronic health condition and the focus of healthcare and research is shifting towards outcomes on the long-term [2–4]. Next to monitoring the health status of chronically ill children, the impact of events such as the COVID-19 pandemic on the physical and mental health of these children should be carefully monitored.

Input from parents and their children has become an essential part of modern pediatric healthcare and parent- or patient-reported outcome measures (PROMs) are increasingly being implemented [5]. The information collected from PROMs can help clinicians to monitor a child’s progress, to guide and adjust treatment, and to improve the quality of value-based healthcare [5, 6]. One of the most frequently used instruments to measure children’s health status (HS) is the Pediatric Quality of Life Inventory (PedsQL™)[7]. The PedsQL has been translated into multiple languages and data have been validated for over 35 translations worldwide [8].

Previous research has shown that adolescent and adult females report a lower HS than their male age peers [9, 10]; it is not inconceivable that this phenomenon goes back to childhood. Previous PedsQL validation studies reported a lower HS in girls in the United Kingdom [11] but no sex differences in the United States [7]. Establishing sex-specific normative values can help improve our understanding of sex differences and, consequently, enable the provision of sex-sensitive healthcare [12, 13].

In the Netherlands, the most recent data from a large sample for the online version of the PedsQL have been collected more than ten years ago [14]. Despite the finding that girls aged 8–12 years reported lower Emotional Functioning than boys [14], sex-specific data were not presented. Next to this, parent proxy-report data in children aged 8–17 years are still lacking in the Netherlands [14]. It is necessary to also obtain proxy data from a large sample as child self-reports regularly need to be substituted by parent proxy-reports for a variety of reasons. For instance, a child may be too young or too ill to complete the questionnaire. Next to this, inclusion of a proxy-report provides a broader view of the child’s health status.

For this study, we collected sex-specific data for both the parent proxy-report and the child self-report online version of the PedsQL. The primary aim of our study was to determine whether the discrepancy in health status among males and females already develops during childhood and adolescence. Secondarily, we assessed agreement between child self-reports and parent proxy-reports and evaluated sociodemographic influences on PedsQL scores.

## Methods

### Participants and procedure

The PedsQL was administered online to healthy Dutch children aged 5–17 years and their parents, between April 2015 and June 2017, using our institutional program for online surveys (LimeSurvey GmbH version 2.06lts, Hamburg, Germany). Participants were recruited through regular primary and secondary schools in different regions of the Netherlands. Children with a parent-reported chronic disease (e.g. asthma, cerebral palsy; according to the 10^th^ revision of the International Statistical Classification of Diseases and Related Health Problems [15]) and/or a mental disorder (e.g. autism spectrum disorder, attention deficit/hyperactivity disorder, anxiety disorder; according to the 5^th^ edition of the Diagnostic and Statistical Manual of Mental Disorders [16]) were excluded from analysis of both proxy-reported and self-reported data. Parents of participating schools had been sent a letter in which the purpose and procedure of the study were explained. All parents, as well as children aged 12 years and older, had been asked to provide informed consent for use of data for study purposes before filling out the online questionnaire. Parent and child responses were anonymously linked by means of personal tokens. The local Medical Ethics Review Board waived approval (‘Medical Research in Human Subjects Act does not apply to this research proposal’; Medical Ethics Committee Erasmus MC; MEC 2015–244).

### Measures

#### PedsQL

The PedsQL assesses HS on four subscales: Physical (8 items), Emotional (5 items), Social (5 items), and School Functioning (5 items). Psychosocial Health is calculated as the mean score of Emotional, Social, and School Functioning. The Total Score is calculated as the mean score of all four subscales. Each item reflects a problem, for example 'problems with running', during the past month. Answers vary from never (= 0) to almost always (= 4) on a 5-point Likert scale. The scoring of each answer is reversed, and rescaled to a 0–100 scale (0 = 100, 4 = 0). Total Scores range from 0–100; higher scores reflect better health status [7]. We administered three age-appropriate versions of the PedsQL in this study: 5–7 years (parent proxy-report), 8–12 years (child self-report and parent proxy-report), and 13–17 years (child self-report and parent proxy-report). The layout of our web-based version of the PedsQL resembled the paper version as much as possible, except that questions were presented per subscale instead of all at once and missing values were not accepted.

#### Sociodemographic questionnaire

Parents were asked to fill out a sociodemographic questionnaire, consisting of three items on own sex, country of birth of both parents (i.e. the Netherlands or another country), and highest completed education: low, middle, or high based on the International Standard Classification of Education (ISCED) 2011 [17]), and four items concerning their child (sex, month and year of birth, presence of a chronic disease, and need for psychological help during the past year).

### Statistical analysis

Continuous sociodemographic variables are presented as median (range) and categorical variables as number (%). Associations between sociodemographic characteristics (i.e. parental sex, level of education, and country of birth) and PedsQL scores were assessed using Mann–Whitney U-tests. To facilitate comparisons with reference data obtained in other populations, PedsQL scores are shown as mean ± standard deviation (SD). Differences in PedsQL scores between boys and girls were compared using t-tests or Mann–Whitney U tests, depending on the distribution of the data (i.e. normally or not normally distributed according to Shapiro–Wilk test). Wilcoxon signed-rank tests and intraclass correlation coefficients (ICCs; two-way mixed model, absolute agreement, single measures) served to compare parent proxy-reports with child self-reports. ICCs were interpreted as poor agreement (< 0.50), moderate agreement (0.50–0.75), good agreement (0.75–0.90), or excellent agreement (> 0.90) [18]. Effect sizes were calculated using Cohen’s *d*. Effect sizes were considered small (0.20), medium (0.50), or large (0.80) [19]. Additionally, we performed linear multivariable regression analyses to assess the relationship between sex of the child, age, and parental educational level (dichotomized into ISCED class Low or Middle/High – based on a one-way ANOVA test) selected as independent variables, and PedsQL scores (each subscale selected as dependent variable). We applied a Bonferroni correction to account for multiple comparisons; as we assessed sociodemographic influences and sex differences for two (child self-reports) or three (parent proxy-reports) different age categories: alpha was set at 0.05/2 = 0.025 or 0.05/3 = 0.017, respectively. Statistical analyses were performed using SPSS V.24.0.

## Results

**Fig. 1:**
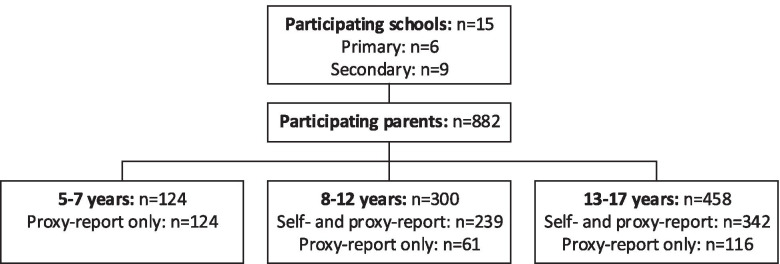
Inclusion flow chart. Regions: West (n = 8), South (n = 6), East (n = 1)

We recruited 882 parents and 581 children from 15 different schools in the Netherlands, who completed the PedsQL (Fig. 1). Sociodemographic characteristics of participants are shown in Table 1.

**Table 1:** Sociodemographic characteristics per age category

	**Age 5–7 years** n = 124	**Age 8–12 years** n = 300	**Age 13–17 years** n = 458
**Child characteristics**			
Age (years)	6.3 (5.0–7.9)	11.0 (8.0–12.9)	14.8 (13.0–17.9)
Sex (boy)	52 (42%)	121 (40%)	221 (48%)
Psychological help	6 (5%)	23 (8%)	29 (6%)
**Parent characteristics**			
Sex (male)	13 (10%)	32 (11%)	73 (16%)
Educational level (ISCED) Low (0–2) Middle (3–4) High (5–8)	12 (10%) 36 (29%) 76 (61%)	26 (9%) 84 (28%) 190 (63%)	38 (8%) 86 (19%) 334 (73%)
Both parents Dutch	106 (85%)	256 (85%)	387 (84%)

Data are presented as median (range) or n (%), *ISCED* International Standard Classification of Education

### Sociodemographic influences

#### Parental sex

We found no effect of parental sex in any age group; PedsQL scores reported by fathers did not differ significantly from those reported by mothers.

#### Level of education

In multivariable linear regression analyses, parental level of education was found only to be predictive of proxy-rated School Functioning in 8–12 year-olds (unstandardized regression coefficient = 8.03, p = 0.006; Additional file 4A), which means that parents with a higher educational level rated the School Functioning of their child in this age group higher than parents with a lower educational level did.

#### Country of birth

In proxy ratings, School Functioning of children with two Dutch parents was rated higher than that of age peers with one Dutch parent and a parent born in a different country in the age categories of 5–7 years (mean difference: 11.53, effect size: 0.65, p = 0.012) and 13–17 years (mean difference: 4.94, effect size: 0.32, p = 0.016). Apart from this, no significant differences in any age category were found with regard to country of birth – neither for parent proxy-reports nor for child self-reports.

#### Age

In multivariable linear regression analyses, an additional effect of age was found in 8–12 year-olds for self-rated School Functioning (unstandardized regression coefficient = 1.38, p = 0.010; Additional file 4B), which means that older children report better School Functioning than younger children in this age group did.

### Sex differences

**Fig. 2:**
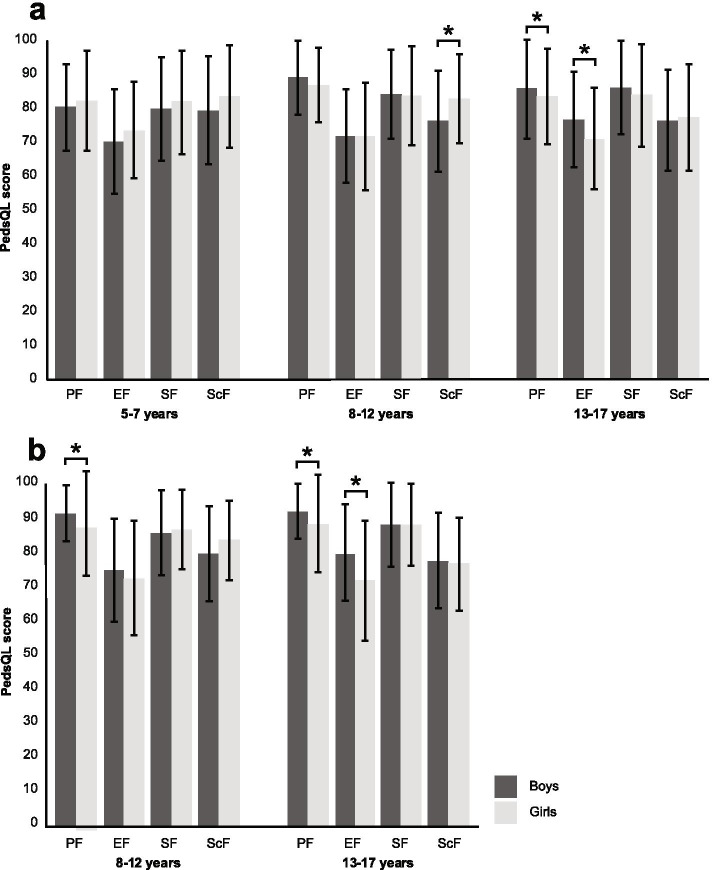
Bar chart showing sex differences per PedsQL subscale. Panel a: parent proxy-report; panel b: child self-report. PF = Physical Functioning; EF = Emotional Functioning; SF = Social Functioning; ScF = School Functioning. Asterisk indicates significance, on a Bonferroni-adjusted significance level of 0.017 (parent proxy-report) or 0.025 (child self-report)

Parents of 5-to-7-year-olds reported similar scores for boys and girls. In the group of 8-to-12-year-olds, parents reported higher scores on School Functioning for girls than for boys (mean difference: 6.56, p < 0.001). Parents of 13-to-17-year-olds reported lower scores on Physical Functioning and Emotional Functioning for girls than for boys (mean differences: 2.14 and 5.79; p = 0.014 and p < 0.001, respectively) (Table 2, Fig. 2, and Additional file 1). Effect sizes ranged from 0.01 to 0.46.

**Table 2:** Sex differences in parent proxy-reports per age category

	**PedsQL scale**	**Mean ± SD**		**Effect size** **Cohen’s *d***	***p* value**
		**Boys (n = 52)**	**Girls (n = 72)**		
**5–7 years**	Physical Functioning	81.61 ± 13.0	83.55 ± 15.0	0.14	0.240 ^a^
	Emotional Functioning	71.35 ± 15.6	74.80 ± 14.4	0.22	0.209 ^b^
	Social Functioning	81.15 ± 15.5	83.19 ± 15.5	0.13	0.417 ^a^
	School Functioning	80.67 ± 16.1	85.00 ± 15.3	0.27	0.100 ^a^
	Psychosocial Health	77.72 ± 12.0	81.00 ± 12.4	0.27	0.136 ^a^
	Total Score	77.96 ± 12.2	80.63 ± 12.0	0.22	0.227 ^b^
		**Boys (n = 121)**	**Girls (n = 179)**		
**8–12 years**	Physical Functioning	90.47 ± 11.0	88.30 ± 11.3	0.19	0.031 ^a^
	Emotional Functioning	72.85 ± 14.0	72.93 ± 16.2	0.01	0.937 ^a^
	Social Functioning	85.54 ± 13.4	84.97 ± 14.8	0.04	0.952 ^a^
	School Functioning	77.52 ± 15.2	84.08 ± 13.3	0.46	< 0.001* ^a^
	Psychosocial Health	78.64 ± 10.8	80.66 ± 11.6	0.18	0.075 ^a^
	Total Score	82.75 ± 9.3	83.35 ± 10.4	0.06	0.393 ^a^
		**Boys (n = 221)**	**Girls (n = 237)**		
**13–17 years**	Physical Functioning	87.13 ± 14.8	84.99 ± 14.3	0.15	0.014 * ^a^
	Emotional Functioning	77.96 ± 14.3	72.17 ± 15.0	0.40	< 0.001* ^a^
	Social Functioning	87.44 ± 14.1	85.17 ± 15.2	0.15	0.067 ^a^
	School Functioning	77.76 ± 15.2	78.59 ± 15.9	0.05	0.409 ^a^
	Psychosocial Health	81.06 ± 11.6	78.64 ± 12.5	0.20	0.037 ^a^
	Total Score	83.17 ± 11.2	80.85 ± 11.5	0.20	0.036 ^a^

*SD* standard deviation. * Bonferroni-adjusted significance level of 0.017 due to stratification by age group. ^a^ Mann–Whitney U test. ^b^ Independent t-test

Girls aged 8–12 years reported lower scores than boys on Physical Functioning (mean difference: 3.09, p = 0.005). Girls aged 13–17 years reported lower scores than boys on Physical Functioning (mean difference: 3.67, p < 0.001), Emotional Functioning (mean difference: 8.11, p < 0.001), and on the Total Score (mean difference 3.26, p = 0.004) (Table 3, Fig. 2, and Additional file 2). Effect sizes ranged from 0.00 to 0.50.

**Table 3:** Sex differences in child self-reports per age category

	**PedsQL scale**	**Mean ± SD**		**Effect size** **Cohen’s *d***	***p* value**
		**Boys (n = 93)**	**Girls (n = 146)**		
**8–12 years**	Physical Functioning	92.47 ± 8.3	89.38 ± 9.5	0.35	0.005* ^a^
	Emotional Functioning	75.59 ± 15.3	73.29 ± 17.0	0.14	0.245 ^a^
	Social Functioning	86.45 ± 12.6	87.60 ± 11.8	0.09	0.529 ^a^
	School Functioning	80.48 ± 14.0	84.42 ± 11.6	0.31	0.042 ^a^
	Psychosocial Health	80.84 ± 10.8	81.77 ± 10.8	0.09	0.541 ^a^
	Total Score	84.89 ± 8.7	84.42 ± 9.5	0.05	0.729 ^a^
		**Boys (n = 160)**	**Girls (n = 182)**		
**13–17 years**	Physical Functioning	93.09 ± 8.1	89.42 ± 10.4	0.39	< 0.001* ^a^
	Emotional Functioning	80.53 ± 14.3	72.42 ± 17.7	0.50	< 0.001* ^a^
	Social Functioning	89.00 ± 12.4	89.04 ± 12.1	0.00	0.824 ^a^
	School Functioning	78.47 ± 14.1	77.39 ± 13.7	0.08	0.482 ^a^
	Psychosocial Health	82.67 ± 10.3	79.62 ± 11.6	0.28	0.028 ^a^
	Total Score	86.29 ± 8.6	83.03 ± 9.8	0.35	0.004* ^a^

*SD* standard deviation. * Bonferroni-adjusted significance level of 0.025 due to stratification by age group. ^a^ Mann–Whitney U test

In multivariable regression analyses, sex of the child was found to be predictive of both proxy- and self-reported School Functioning in 8–12 year-olds (coefficient = -7.18 and -3.92, p < 0.001 and p = 0.022, respectively), indicating *higher* scores for boys on School Functioning in this age group. In the group of 13-to-17-year olds, sex of the child was significantly predictive of self-reported Physical Functioning (coefficient = 3.59, p < 0.001), proxy- and self-reported Emotional Functioning (coefficient = 5.75 and 8.13, respectively, both p < 0.001), self-reported Psychosocial Health (coefficient = 3.09, p = 0.011), and of the self-reported Total Score (coefficient = 3.27, p = 0.001; Additional file 4A and B). This means that in 13–17-year olds, scores of girls and parents of girls were *lower* than scores of boys and parents of boys on these scales.

### Differences between child self-report and parent proxy-report

In the age category of 8–12 years, parent proxy-reports were comparable to child self-reports (Table 4). Children aged 13–17 years reported higher scores than their parents on Physical Functioning (mean difference: 4.03, p < 0.001), and on the Total Score (mean difference: 1.57, p = 0.017). Effect sizes ranged from 0.00 to 0.34. Agreement between child self-reports and parent proxy-reports was poor to moderate; ICCs ranged from 0.34 (Physical Functioning in 13-to-17-year-olds) to 0.58 (Emotional Functioning in 8-to-12-year-olds). A visual representation of agreement on PedsQL Total Scores between parents and children is given in Additional file 3.

**Table 4:** Differences between child self-report and parent proxy-report per age category

	**PedsQL scale**	**Mean ± SD**		**ICC (95% CI)**	**Effect size** **Cohen’s *d***	***p* value**
		**Child self-report (n = 239)**	**Parent proxy-report (n = 239)**			
**8–12 years**	Physical Functioning	90.59 ± 9.2	90.23 ± 10.9	0.48 (0.38–0.57)	0.04	0.667
	Emotional Functioning	74.18 ± 16.3	73.66 ± 15.3	0.58 (0.49–0.66)	0.03	0.570
	Social Functioning	87.15 ± 12.1	85.96 ± 13.8	0.47 (0.37–0.57)	0.09	0.370
	School Functioning	82.89 ± 12.7	82.95 ± 14.1	0.55 (0.45–0.63)	0.00	0.828
	Psychosocial Health	81.41 ± 10.8	80.86 ± 11.1	0.55 (0.46–0.63)	0.05	0.435
	Total Score	84.60 ± 9.2	84.12 ± 9.5	0.55 (0.45–0.63)	0.05	0.598
		**Child self-report (n = 342)**	**Parent proxy-report (n = 342)**			
**13–17 years**	Physical Functioning	91.14 ± 9.6	87.11 ± 13.5	0.34 (0.24–0.44)	0.34	< 0.001*
	Emotional Functioning	76.21 ± 16.7	75.77 ± 15.0	0.48 (0.39–0.56)	0.03	0.338
	Social Functioning	89.02 ± 12.2	87.08 ± 13.9	0.42 (0.33–0.50)	0.15	0.042
	School Functioning	77.89 ± 13.9	79.49 ± 14.9	0.45 (0.36–0.53)	0.11	0.048
	Psychosocial Health	81.04 ± 11.1	80.78 ± 11.7	0.48 (0.39–0.55)	0.02	0.892
	Total Score	84.55 ± 9.4	82.98 ± 10.6	0.48 (0.40–0.56)	0.16	0.017*

*SD* standard deviation, *ICC* intraclass correlation coefficient, *CI* confidence interval. * Bonferroni-adjusted significance level of 0.025 due to stratification by age group, using Wilcoxon signed-rank test

## Discussion

We collected online data for the PedsQL in a school-based sample of 882 parents and 581 healthy children. Statistically significant sex differences were found from the age of 8 years onwards; girls generally had lower scores than boys, except for parent-reported School Functioning at age 8–12 years, which was higher in girls. Sex differences remained present after correcting for age and parental educational level, suggesting that boys and girls have different HS patterns that need to be assessed separately. Parents of children aged 13–17 years rated their child’s Physical Functioning lower than did the children themselves.

Sex differences in HS have been well reported among adults. Women reporting lower scores than men has been demonstrated not only for the PedsQL [9, 10], but also for other HS instruments across the world [20–22]. Our finding that sex differences in HS already emerge during childhood is supported by previous studies in clinical groups [14, 23–28]. Although these studies unanimously concluded that girls report lower scores than boys, their results varied with regard to the specific subscales. Some studies reported sex differences in Emotional Functioning only [14, 25], whereas others showed differences on a larger variety of subscales and on the Total Score [23, 24, 26–28].

The agreement between child self-reported and parent proxy-reported HS differs between studies. While some studies found that parents reported their child’s HS higher than did the child itself [29], other studies reported opposite findings [30, 31], or found differences in both directions – depending on the subscale [25, 32]. To make things even more complicated, previous research in children with chronic conditions has shown that sex – either that of the parent or the child – may affect parent–child agreement. Ooi et al., for instance, reported that parent–child agreement in children with obesity was higher with mothers than with fathers as proxies [31]. Blake et al. studied adolescents with sickle cell disease and found that parents of girls reported a higher HS than did the girls themselves, whereas parents of boys reported a lower HS than did the boys themselves [32]. One should keep in mind that a study’s conclusion might also depend on the way the data are assessed and interpreted: although we barely found any statistically significant differences between parent proxy-reports and child self-reports, ICCs showed poor to moderate agreement between parents and their children. Cross-informant discrepancies are a common finding; not only for the PedsQL, but also for other instruments such as the Child Behavior Checklist and the Youth Self Report [33]. These differences may indicate that both informants make unique contributions to a view of children’s HS.

Discrepancies between studies on HS with regard to sex differences or parent–child agreement can have various causes. HS may be affected by country of residence [27, 28], differences over time [34], socioeconomic status [28], physical and mental conditions [14, 35], a child’s age [28], and presumably many other factors. These factors need to be taken into account as much as possible when interpreting a study’s results. For this reason, up-to-date, country-specific data are essential to make valid inferences about a child’s HS. We chose not to present any cut-off points, but rather would recommend longitudinal follow-up of children’s PedsQL scores, which would alert to possible differences between time points and/or between child self-report and parent proxy-reports.

One of the strengths of our study is its large sample size, allowing us to present valid sex-specific data for both parent proxy-reported and child self-reported HS. Second, we included most regions of our country to improve the study’s representativeness of the general Dutch population. Several limitations need to be taken into account. First, as we did not collect data of non-participants, we compared our sociodemographic data to those of the general Dutch population. The proportion of participants with a Dutch background in our study was slightly higher than that of the total population in 2015 (85% versus 78% [36]). This finding is in line with the previous Dutch normative study [14], and may be explained by the fact that parents with insufficient understanding of the Dutch language could not participate. Furthermore, a relatively high proportion of participants had a high educational level (68% versus 43% in the general Dutch population aged 25–45 years [37]). This finding is not uncommon in similar studies [14, 38], but needs to be taken into account when interpreting these data. Second, we did not evaluate child self-reports in 5–7 year-olds. Based on our own experience within the infrastructure of a longitudinal follow-up program for children with congenital anatomical anomalies we decided to refrain from using the self-reports for 5–7 years. Our decision is justified by the recent publication of Conijn and coworkers [39]. Third, fathers were underrepresented, as more than 80% of proxy-reports were filled out by mothers. This is a common finding as well [7, 14]. Nevertheless, as we found no effect of parental sex in any age group, we presume that this underrepresentation has had little effect on our results.

## Conclusions

Our study is the first to report sex-specific PedsQL data for a large school-based sample of the Dutch population. Girls generally had lower PedsQL scores than boys, both in parent proxy-reports and in child self-reports. We recommend to apply sex-specific data when assessing a child’s HS using the PedsQL. Taking into account sex, a fairer distinction can be made between normal and impaired HS. Sex-specific data would be more appropriate to study differences between boys and girls when it comes to the consequences of a disease on HS.

Agreement between parent proxy-reports and child self-reports was poor to moderate; children reported slightly higher scores than their parents, except for School Functioning. We therefore recommend to use both parent and child perspectives to get a complete picture and to address potential differences.

## Supplementary Information

**Additional file 1.** Parent proxy-reports: Normative data, including medians and interquartile ranges.

**Additional file 2.** Child self-reports: Normative data, including medians and interquartile ranges.

**Additional file 3.** Scatter plot showing agreement on PedsQL Total Scores between parent proxy-report and child self-report.

**Additional file 4: Table S1.** Multivariable linear regression analyses showing significant predictors of the PedsQL scales; parent proxy-reports per age category** Table S2.** Multivariable linear regression analyses showing significant predictors of the PedsQL scales; child self-reports per age category.

## Data Availability

Anonymized data are available upon reasonable request by contacting the corresponding author.

## Abbreviations

HS: Health status
ICCs: Intraclass correlation coefficients
ISCED: International Standard Classification of Education
PedsQL: Pediatric Quality of Life Inventory
PROMs: Patient-reported outcome measures
SD: Standard deviation
