# Supplementary material for: Sex differences in children's health status as measured by the Pediatric Quality of Life Inventory (PedsQL)™: cross-sectional findings from a large school-based sample in the Netherlands
Source: BMC Pediatr. 2021 Dec 18;21:580. doi: 10.1186/s12887-021-03059-3 (PMC8683815; doi:10.1186/s12887-021-03059-3)
Supplement: Supplementary file 1 — Additional file 1. Parent proxy-reports: Normative data, including medians and interquartile ranges. [file 12887_2021_3059_MOESM1_ESM.docx]

**Additional file 1.** Parent proxy-reports: Normative data, including medians and interquartile ranges

|  | PedsQL scale | Mean ± SD | | Median (IQR) | |
| --- | --- | --- | --- | --- | --- |
| 5-7 years |  | **Boys (n=52)** | **Girls (n=72)** | **Boys (n=52)** | **Girls (n=72)** |
|  | Physical Functioning | 81.61 ± 13.0 | 83.55 ± 15.0 | 81.25 (75.00-90.63) | 87.50 (78.13-96.09) |
|  | Emotional Functioning | 71.35 ± 15.6 | 74.80 ± 14.4 | 72.50 (60.00-83.75) | 75.00 (65.00-85.00) |
|  | Social Functioning | 81.15 ± 15.5 | 83.19 ± 15.5 | 85.00 (70.00-98.75) | 85.00 (71.25-100.00) |
|  | School Functioning | 80.67 ± 16.1 | 85.00 ± 15.3 | 80.00 (75.00-95.00) | 87.50 (75.00-100.00) |
|  | Psychosocial Functioning | 77.72 ± 12.0 | 81.00 ± 12.4 | 78.33 (68.75-86.25) | 81.67 (70.00-93.33) |
|  | Total Score | 77.96 ± 12.2 | 80.63 ± 12.0 | 78.10 (68.82-86.61) | 79.22 (73.27-90.99) |
| 8-12 years |  | **Boys (n=121)** | **Girls (n=179)** | **Boys (n=121)** | **Girls (n=179)** |
|  | Physical Functioning | 90.47 ± 11.0 | 88.30 ± 11.3 | 93.75 (84.38-100.00) | 90.63 (84.38-96.88) |
|  | Emotional Functioning | 72.85 ± 14.0 | 72.93 ± 16.2 | 75.00 (65.00-82.50) | 75.00 (60.00-90.00) |
|  | Social Functioning | 85.54 ± 13.4 | 84.97 ± 14.8 | 90.00 (75.00-100.00) | 90.00 (75.00-100.00) |
|  | School Functioning | 77.52 ± 15.2 | 84.08 ± 13.3 | 75.00 (65.00-90.00) | 85.00 (75.00-95.00) |
|  | Psychosocial Functioning | 78.64 ± 10.8 | 80.66 ± 11.6 | 78.33 (71.67-86.67) | 81.67 (73.33-90.00) |
|  | Total Score | 82.75 ± 9.3 | 83.35 ± 10.4 | 83.70 (77.17-90.22) | 84.78 (77.17-91.30) |
|  |  | **Boys (n=221)** | **Girls (n=237)** | **Boys (n=221)** | **Girls (n=237)** |
| 13-17 years | Physical Functioning | 87.13 ± 14.8 | 84.99 ± 14.3 | 90.63 (81.25-96.88) | 90.63 (79.69-93.75) |
|  | Emotional Functioning | 77.96 ± 14.3 | 72.17 ± 15.0 | 80.00 (70.00-90.00) | 70.00 (60.00-85.00) |
|  | Social Functioning | 87.44 ± 14.1 | 85.17 ± 15.2 | 95.00 (80.00-100.00) | 90.00 (75.00-100.00) |
|  | School Functioning | 77.76 ± 15.2 | 78.59 ± 15.9 | 80.00 (70.00-90.00) | 80.00 (67.50-90.00) |
|  | Psychosocial Functioning | 81.06 ± 11.6 | 78.64 ± 12.5 | 81.67 (75.00-90.00) | 78.33 (71.67-88.33) |
|  | Total Score | 83.17 ± 11.2 | 80.85 ± 11.5 | 84.78 (77.17-91.30) | 82.61 (73.91-89.13) |

SD: standard deviation; IQR: interquartile range.
