# Supplementary material for: Sex differences in children's health status as measured by the Pediatric Quality of Life Inventory (PedsQL)™: cross-sectional findings from a large school-based sample in the Netherlands
Source: BMC Pediatr. 2021 Dec 18;21:580. doi: 10.1186/s12887-021-03059-3 (PMC8683815; doi:10.1186/s12887-021-03059-3)
Supplement: Supplementary file 2 — Additional file 2. Child self-reports: Normative data, including medians and interquartile ranges. [file 12887_2021_3059_MOESM2_ESM.docx]

**Additional file 2.** Child self-reports: Normative data, including medians and interquartile ranges

|  | PedsQL scale | Mean ± SD | | Median (IQR) | |
| --- | --- | --- | --- | --- | --- |
| 8-12 years |  | **Boys (n=93)** | **Girls (n=146)** | **Boys (n=93)** | **Girls (n=146)** |
|  | Physical Functioning | 92.47 ± 8.3 | 89.38 ± 9.5 | 93.75 (90.63-100.00) | 90.63 (84.38-96.88) |
|  | Emotional Functioning | 75.59 ± 15.3 | 73.29 ± 17.0 | 80.00 (65.00-85.00) | 75.00 (60.00-85.00) |
|  | Social Functioning | 86.45 ± 12.6 | 87.60 ± 11.8 | 90.00 (75.00-100.00) | 90.00 (80.00-100.00) |
|  | School Functioning | 80.48 ± 14.0 | 84.42 ± 11.6 | 80.00 (75.00-90.00) | 85.00 (75.00-95.00) |
|  | Psychosocial Functioning | 80.84 ± 10.8 | 81.77 ± 10.8 | 81.67 (75.00-88.33) | 83.33 (76.67-90.00) |
|  | Total Score | 84.89 ± 8.7 | 84.42 ± 9.5 | 85.87 (80.44-91.30) | 85.87 (79.35-91.30) |
|  |  | **Boys (n=160)** | **Girls (n=182)** | **Boys (n=160)** | **Girls (n=182)** |
| 13-17 years | Physical Functioning | 93.09 ± 8.1 | 89.42 ± 10.4 | 95.31 (90.63-100.00) | 93.75 (84.38-96.88) |
|  | Emotional Functioning | 80.53 ± 14.3 | 72.42 ± 17.7 | 80.00 (70.00-90.00) | 75.00 (60.00-85.00) |
|  | Social Functioning | 89.00 ± 12.4 | 89.04 ± 12.1 | 95.00 (80.00-100.00) | 92.50 (80.00-100.00) |
|  | School Functioning | 78.47 ± 14.1 | 77.39 ± 13.7 | 80.00 (70.00-90.00) | 80.00 (70.00-90.00) |
|  | Psychosocial Functioning | 82.67 ± 10.3 | 79.62 ± 11.6 | 81.67 (75.00-91.67) | 80.00 (71.67-90.00) |
|  | Total Score | 86.29 ± 8.6 | 83.03 ± 9.8 | 85.87 (80.43-93.48) | 83.70 (76.09-90.49) |

SD: standard deviation; IQR: interquartile range.
