# Supplementary material for: Sex differences in children's health status as measured by the Pediatric Quality of Life Inventory (PedsQL)™: cross-sectional findings from a large school-based sample in the Netherlands
Source: BMC Pediatr. 2021 Dec 18;21:580. doi: 10.1186/s12887-021-03059-3 (PMC8683815; doi:10.1186/s12887-021-03059-3)
Supplement: Supplementary file 3 — Additional file 3. Scatter plot showing agreement on PedsQL Total Scores between parent proxy-report and child self-report. [file 12887_2021_3059_MOESM3_ESM.docx]

**Additional file 3.** Scatter plot showing agreement on PedsQL Total Scores between parent proxy-report and child self-report


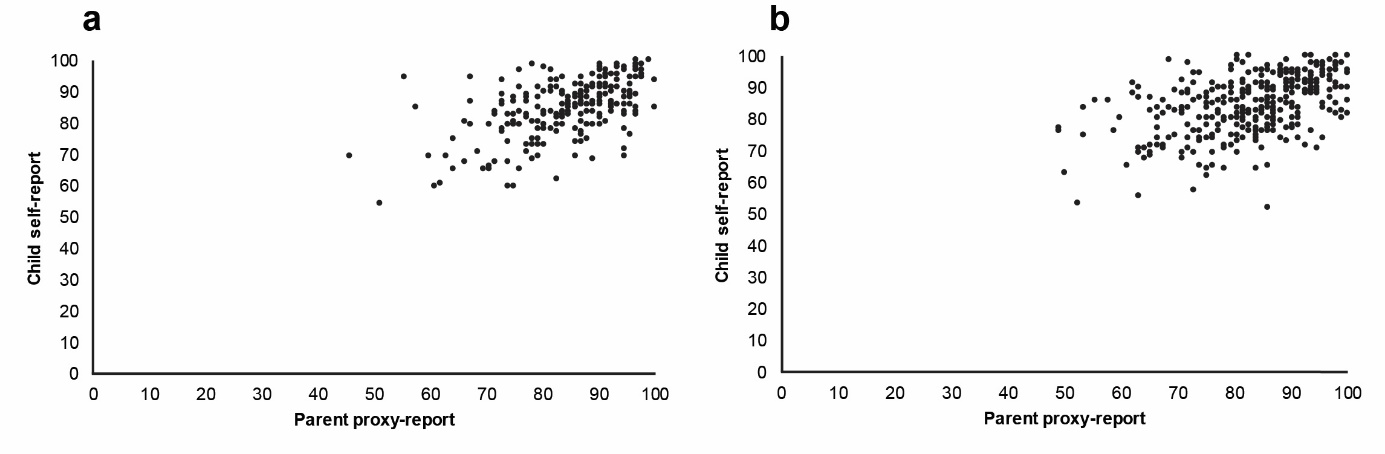


Panel a: 8–to-12-year-olds; panel b: 13–to-17-year-olds
