# Supplementary material for: Sex differences in children's health status as measured by the Pediatric Quality of Life Inventory (PedsQL)™: cross-sectional findings from a large school-based sample in the Netherlands
Source: BMC Pediatr. 2021 Dec 18;21:580. doi: 10.1186/s12887-021-03059-3 (PMC8683815; doi:10.1186/s12887-021-03059-3)
Supplement: Supplementary file 4 — Additional file 4: Table S1. Multivariable linear regression analyses showing significant predictors of the PedsQL scales; parent proxy-reports per age category Table S2. Multivariable linear regression analyses showing significant predictors of the PedsQL scales; child self-reports per age category. [file 12887_2021_3059_MOESM4_ESM.zip › BMCPed_Additional file 4 v2.docx]

**Additional file 4.**

**Table 1.** Multivariable linear regression analyses showing significant predictors of the PedsQL scales; parent proxy-reports per age category

|  |  | Independent variables | | | | | |
| --- | --- | --- | --- | --- | --- | --- | --- |
|  |  | **Sex** | | **Age** | | **Parental educational level (ISCED)** | |
|  | **Dependent variables (PedsQL scales)** | B | *p* value | B | *p* value | B | *p* value |
| 5-7 years | Physical Functioning | -1.35 | 0.605 | 2.16 | 0.180 | 3.24 | 0.453 |
|  | Emotional Functioning | -3.07 | 0.270 | 1.38 | 0.422 | 2.04 | 0.657 |
|  | Social Functioning | -1.73 | 0.545 | 0.26 | 0.883 | 7.97 | 0.093 |
|  | School Functioning | -4.78 | 0.095 | -3.12 | 0.077 | 7.74 | 0.102 |
|  | Psychosocial Health | -3.19 | 0.158 | -0.50 | 0.721 | 5.91 | 0.114 |
|  | Total Score | -2.34 | 0.296 | 0.83 | 0.548 | 4.79 | 0.196 |
| 8-12 years | Physical Functioning | 1.67 | 0.214 | 0.60 | 0.172 | 1.95 | 0.400 |
|  | Emotional Functioning | 0.28 | 0.880 | -0.27 | 0.649 | -3.67 | 0.250 |
|  | Social Functioning | 0.02 | 0.99 | 0.83 | 0.132 | 2.26 | 0.441 |
|  | School Functioning | -7.18 | <0.001* | 0.26 | 0.628 | 8.03 | 0.006* |
|  | Psychosocial Health | -2.30 | 0.091 | 0.28 | 0.531 | 2.21 | 0.345 |
|  | Total Score | -0.92 | 0.444 | 0.39 | 0.319 | 2.12 | 0.304 |
| 13-17 years | Physical Functioning | 2.20 | 0.107 | -0.14 | 0.783 | 5.42 | 0.028 |
|  | Emotional Functioning | 5.75 | <0.001* | 0.12 | 0.814 | -2.22 | 0.374 |
|  | Social Functioning | 2.28 | 0.099 | -0.10 | 0.847 | -2.46 | 0.325 |
|  | School Functioning | -0.66 | 0.654 | -0.80 | 0.153 | 0.99 | 0.709 |
|  | Psychosocial Health | 2.46 | 0.031 | -0.26 | 0.550 | -1.23 | 0.549 |
|  | Total Score | 2.37 | 0.027 | -0.22 | 0.591 | 1.09 | 0.573 |

Sex was categorized as girl (0) or boy (1); parental educational level was categorized as ISCED low (0) or middle/high (1). * Bonferroni-adjusted significance level of 0.017 due to stratification by age group. ISCED: International Standard Classification of Education.

**Table 2.** Multivariable linear regression analyses showing significant predictors of the PedsQL scales; child self-reports per age category

|  |  | Independent variables | | | | | |
| --- | --- | --- | --- | --- | --- | --- | --- |
|  |  | **Sex** | | **Age** | | **Parental educational level** | |
|  | **Dependent variables (PedsQL scales)** | B | *p* value | B | *p* value | B | *p* value |
| **8-12 years** | Physical Functioning | 2.70 | 0.027 | 0.82 | 0.042 | 0.11 | 0.961 |
|  | Emotional Functioning | 2.09 | 0.343 | 0.89 | 0.219 | -4.48 | 0.278 |
|  | Social Functioning | -1.88 | 0.246 | 1.38 | 0.010* | 1.71 | 0.573 |
|  | School Functioning | -3.92 | 0.022* | -0.39 | 0.485 | 3.66 | 0.252 |
|  | Psychosocial Health | -1.24 | 0.398 | 0.63 | 0.193 | 0.30 | 0.914 |
|  | Total Score | 0.13 | 0.916 | 0.69 | 0.091 | 0.23 | 0.921 |
| **13-17 years** | Physical Functioning | 3.59 | <0.001* | 0.49 | 0.217 | 2.20 | 0.243 |
|  | Emotional Functioning | 8.13 | <0.001* | -0.22 | 0.746 | -1.89 | 0.564 |
|  | Social Functioning | -0.09 | 0.949 | 0.08 | 0.879 | -1.86 | 0.451 |
|  | School Functioning | 1.24 | 0.415 | -0.56 | 0.338 | 2.33 | 0.405 |
|  | Psychosocial Health | 3.09 | 0.011* | -0.24 | 0.614 | -0.47 | 0.832 |
|  | Total Score | 3.27 | 0.001* | 0.02 | 0.966 | 0.46 | 0.806 |

Sex was categorized as girl (0) or boy (1); parental educational level was categorized as ISCED low (0) or middle/high (1). * Bonferroni-adjusted significance level of 0.025 due to stratification by age group. ISCED: International Standard Classification of Education.
