# Supplementary material for: Sex differences in children's health status as measured by the Pediatric Quality of Life Inventory (PedsQL)™: cross-sectional findings from a large school-based sample in the Netherlands
Source: BMC Pediatr. 2021 Dec 18;21:580. doi: 10.1186/s12887-021-03059-3 (PMC8683815; doi:10.1186/s12887-021-03059-3)
Supplement: Supplementary file 4 — Additional file 4: Table S1. Multivariable linear regression analyses showing significant predictors of the PedsQL scales; parent proxy-reports per age category Table S2. Multivariable linear regression analyses showing significant predictors of the PedsQL scales; child self-reports per age category. [file 12887_2021_3059_MOESM4_ESM.zip › BMCPed_Additional file 4 v3.docx]

**Additional file 4A.** Multivariable linear regression analyses showing significant predictors of the PedsQL scales; parent proxy-reports per age category

|  |  | Independent variables | | | | | | | | | | | |
| --- | --- | --- | --- | --- | --- | --- | --- | --- | --- | --- | --- | --- | --- |
|  |  | **Sex** |  | | | **Age** |  | | | **Parental educational level (ISCED)** | |  | |
|  | **Dependent variables (PedsQL scales)** | Coef | *95% CI*  *Lower* | *95% CI*  *Upper* | *p* value | Coef | *95% CI*  *Lower* | *95% CI*  *Upper* | *p* value | Coef | *95% CI*  *Lower* | *95% CI*  *Upper* | *p* value |
| 5-7 years | Physical Functioning | -1.35 | -6.51 | 3.80 | 0.605 | 2.16 | -1.01 | 5.34 | 0.180 | 3.24 | -5.28 | 11.76 | 0.453 |
|  | Emotional Functioning | -3.07 | -8.56 | 2.42 | 0.270 | 1.38 | -2.01 | 4.76 | 0.422 | 2.04 | -7.04 | 11.11 | 0.657 |
|  | Social Functioning | -1.73 | -7.36 | 3.91 | 0.545 | 0.26 | -3.21 | 3.73 | 0.883 | 7.97 | -1.35 | 17.28 | 0.093 |
|  | School Functioning | -4.78 | -10.40 | 0.85 | 0.095 | -3.12 | -6.58 | 0.35 | 0.077 | 7.74 | -1.56 | 17.03 | 0.102 |
|  | Psychosocial Health | -3.19 | -7.64 | 1.26 | 0.158 | -0.50 | -3.24 | 2.25 | 0.721 | 5.91 | -1.45 | 13.27 | 0.114 |
|  | Total Score | -2.34 | -6.75 | 2.07 | 0.296 | 0.83 | -1.89 | 3.54 | 0.548 | 4.79 | -2.50 | 12.08 | 0.196 |
| 8-12 years | Physical Functioning | 1.67 | -0.97 | 4.31 | 0.214 | 0.60 | -0.26 | 1.45 | 0.172 | 1.95 | -2.60 | 6.50 | 0.400 |
|  | Emotional Functioning | 0.28 | -3.53 | 3.91 | 0.880 | -0.27 | -1.45 | 0.90 | 0.649 | -3.67 | -9.92 | 2.59 | 0.250 |
|  | Social Functioning | 0.02 | -3.33 | 3.37 | 0.99 | 0.83 | -0.25 | 1.92 | 0.132 | 2.26 | -3.51 | 8.04 | 0.441 |
|  | School Functioning | -7.18 | -10.49 | -3.88 | <0.001* | 0.26 | -0.81 | 1.33 | 0.628 | 8.03 | 2.34 | 13.72 | 0.006* |
|  | Psychosocial Health | -2.30 | -4.96 | 0.37 | 0.091 | 0.28 | -0.59 | 1.14 | 0.531 | 2.21 | -2.39 | 6.81 | 0.345 |
|  | Total Score | -0.92 | -3.27 | 1.44 | 0.444 | 0.39 | -0.38 | 1.15 | 0.319 | 2.12 | -1.93 | 6.17 | 0.304 |
| 13-17 years | Physical Functioning | 2.20 | -0.48 | 4.87 | 0.107 | -0.14 | -1.16 | 0.87 | 0.783 | 5.42 | 0.59 | 10.25 | 0.028 |
|  | Emotional Functioning | 5.75 | 3.04 | 8.46 | <0.001* | 0.12 | -0.91 | 1.15 | 0.814 | -2.22 | -7.11 | 2.68 | 0.374 |
|  | Social Functioning | 2.28 | -0.43 | 4.99 | 0.099 | -0.10 | -1.13 | 0.93 | 0.847 | -2.46 | -7.35 | 2.44 | 0.325 |
|  | School Functioning | -0.66 | -3.53 | 2.22 | 0.654 | -0.80 | -1.89 | 0.30 | 0.153 | 0.99 | -4.20 | 6.18 | 0.709 |
|  | Psychosocial Health | 2.46 | 0.23 | 4.69 | 0.031 | -0.26 | -1.10 | 0.59 | 0.550 | -1.23 | -5.26 | 2.80 | 0.549 |
|  | Total Score | 2.37 | 0.28 | 4.46 | 0.027 | -0.22 | -1.01 | 0.58 | 0.591 | 1.09 | -2.69 | 4.86 | 0.573 |

Sex was categorized as girl (0) or boy (1); parental educational level was categorized as ISCED low (0) or middle/high (1). * Bonferroni-adjusted significance level of 0.017 due to stratification by age group. ISCED: International Standard Classification of Education.

**Additional file 4B.** Multivariable linear regression analyses showing significant predictors of the PedsQL scales; child self-reports per age category

|  |  | Independent variables | | | | | | | | | | | |
| --- | --- | --- | --- | --- | --- | --- | --- | --- | --- | --- | --- | --- | --- |
|  |  | **Sex** |  | | | **Age** |  | | | **Parental educational level (ISCED)** | |  | |
|  | **Dependent variables (PedsQL scales)** | Coef | *95% CI*  *Lower* | *95% CI*  *Upper* | *p* value | Coef | *95% CI*  *Lower* | *95% CI*  *Upper* | *p* value | Coef | *95% CI*  *Lower* | *95% CI*  *Upper* | *p* value |
| 8-12 years | Physical Functioning | 2.70 | 0.30 | 5.09 | 0.027 | 0.82 | 0.03 | 1.61 | 0.042 | 0.11 | -4.38 | 4.61 | 0.961 |
|  | Emotional Functioning | 2.09 | -2.25 | 6.42 | 0.343 | 0.89 | -0.53 | 2.31 | 0.219 | -4.48 | -12.61 | 3.64 | 0.278 |
|  | Social Functioning | -1.88 | -5.07 | 1.31 | 0.246 | 1.38 | 0.33 | 2.42 | 0.010* | 1.71 | -4.26 | 7.69 | 0.573 |
|  | School Functioning | -3.92 | -7.26 | -0.57 | 0.022* | -0.39 | -1.49 | 0.71 | 0.485 | 3.66 | -2.62 | 9.93 | 0.252 |
|  | Psychosocial Health | -1.24 | -4.11 | 1.64 | 0.398 | 0.63 | -0.32 | 1.57 | 0.193 | 0.30 | -5.10 | 5.69 | 0.914 |
|  | Total Score | 0.13 | -2.31 | 2.58 | 0.916 | 0.69 | -0.11 | 1.50 | 0.091 | 0.23 | -4.35 | 4.82 | 0.921 |
| 13-17 years | Physical Functioning | 3.59 | 1.59 | 5.60 | <0.001* | 0.49 | -0.29 | 1.27 | 0.217 | 2.20 | -1.50 | 5.91 | 0.243 |
|  | Emotional Functioning | 8.13 | 4.65 | 11.61 | <0.001* | -0.22 | -1.57 | 1.13 | 0.746 | -1.89 | -8.30 | 4.53 | 0.564 |
|  | Social Functioning | -0.09 | -2.71 | 2.54 | 0.949 | 0.08 | -0.94 | 1.10 | 0.879 | -1.86 | -6.71 | 2.99 | 0.451 |
|  | School Functioning | 1.24 | -1.74 | 4.22 | 0.415 | -0.56 | -1.72 | 0.59 | 0.338 | 2.33 | -3.17 | 7.82 | 0.405 |
|  | Psychosocial Health | 3.09 | 0.73 | 5.46 | 0.011* | -0.24 | -1.15 | 0.68 | 0.614 | -0.47 | -4.84 | 3.90 | 0.832 |
|  | Total Score | 3.27 | 1.29 | 5.25 | 0.001* | 0.02 | -0.75 | 0.79 | 0.966 | 0.46 | -3.20 | 4.12 | 0.806 |

Sex was categorized as girl (0) or boy (1); parental educational level was categorized as ISCED low (0) or middle/high (1). * Bonferroni-adjusted significance level of 0.025 due to stratification by age group. ISCED: International Standard Classification of Education.
